# Effects of high-protein diets on the cardiometabolic factors and reproductive hormones of women with polycystic ovary syndrome: a systematic review and meta-analysis

**DOI:** 10.1038/s41387-024-00263-9

**Published:** 2024-02-29

**Authors:** Fang Wang, Pan Dou, Wei Wei, Peng Ju Liu

**Affiliations:** 1 Department of Clinical Nutrition, Peking Union Medical College Hospital (PUMCH), Chinese Academy of Medical Sciences (CAMS) and Peking Union Medical College (PUMC), Dongdansantiao, Dongcheng District, 100730 Beijing, China; 2 Department of Clinical Nutrition, Peking University First Hospital, No.7 Xishiku Dajie, Xicheng District, 100034 Beijing, China

**Keywords:** Obesity, Nutrition

## Abstract

The optimal dietary regimen for polycystic ovary syndrome (PCOS) has not been identified. High-protein diets (HPDs) are effective for weight control in individuals with metabolic abnormalities, but no systematic meta-analyses have yet summarised the effects of HPDs on PCOS. Seven electronic databases were searched from inception to 30 April 2023, and studies comparing the effects of HPDs and other diets on the anthropometrics, metabolic factors, and hormonal profiles for PCOS were identified. Data were pooled using random-effects models and expressed as weighted mean differences and 95% confidence intervals. The risk of bias was assessed by Cochrane Collaboration tool. Eight trials involving 300 women with PCOS were included. Compared with isocaloric balanced diets (BDs), HPDs significantly reduced fasting insulin (−2.69 μIU/mL, 95% CI [−3.81, −1.57], *P* < 0.0001, *I*^2^ = 46%) and homoeostatic model assessment for insulin resistance (HOMA-IR−0.41, 95% CI [−0.80, −0.02], *P* = 0.04, *I*^2^ = 94%) in women with PCOS. However, HPDs and BDs had comparable effects on weight loss, abdominal adiposity, lipid profiles, and reproductive hormones (all *P* ≥ 0.05). HPDs may benefit women with PCOS in terms of improving insulin resistance, supporting for their use as one of the dietary management options for PCOS, however further RCTs in larger and broader settings are required to confirm these observations and investigate the mechanism behind it.

## Introduction

Polycystic ovary syndrome (PCOS), a syndrome characterized by hyperandrogenism, menstrual irregularities, and polycystic ovarian morphology [1], is a common endocrine disorder and a primary cause of anovulation in up to 18% of women of reproductive age. Women with PCOS often exhibit metabolic abnormalities, including insulin resistance (IR), hyperinsulinaemia, and obesity. They are also at an increased risk of developing metabolic syndrome and type 2 diabetes mellitus (T2DM) [2, 3]. IR and hyperinsulinaemia are key pathophysiological factors linked to a series of metabolic and reproductive disorders in women with PCOS [4] and can be exacerbated by overweight or obesity [5]. These conditions affect ovarian function by interacting with gonadotropins, which results in the overproduction of ovarian androgen and prevents ovulation [6].

Weight loss is one of the primary therapies for PCOS. For patients with IR or hyperandrogenism, even a modest weight loss of 5% may have positive effects, such as restoring their regular menses and improving their response to ovulation-induction and fertility medications [7–9]. According to the International Evidence-Based Guidelines for the Assessment and Management of Polycystic Ovary Syndrome, lifestyle modifications such as dietary interventions are recommended as first-line therapy for managing the metabolic complications of PCOS [10]. However, the success and sustainability of weight loss diets have been the subject of debate. According to the literature, women with PCOS tend to be obese [2], do not fully comply with energy-restricted diets [11], and have difficulty in maintaining their weight after weight loss [12, 13], which may be attributed to psychosocial, physiological, or appetite regulatory factors [14, 15]. Therefore, understanding the most useful types and components of diets is essential for the success and sustainability of management strategies that target healthy pregnancies and lifelong health among women with PCOS. Energy restriction, intermittent fasting, high-protein diets (HPDs), low-glycemic index diets, and Mediterranean diets are some of the most effective approaches for weight loss. Of these approaches, HPDs are considered the most effective [16–18], especially their function in improving IR [18].

In terms of muscle and body composition, women with PCOS have a smaller amount of lean body tissue than that of healthy women [19]. Muscles are a crucial endocrine organ. Inadequate muscle mass may reduce the number of insulin receptors and affect the metabolism of glucose and lipids [20]. HPDs can increase muscle mass, thereby improving the control of blood glucose, lipids, and IR. A study indicated that inflammation is one of the most crucial yet overlooked risk factors for PCOS [21], and high-protein intake and improved muscle mass can help improve the inflammatory status of the body [22].

HPDs are widely used for weight control. Although studies have investigated the use of HPDs for patients with PCOS, their results have been inconsistent. Therefore, we conducted a systematic review and meta-analysis to investigate whether HPDs are useful for improving IR, body weight, and glucolipid metabolism in women with PCOS. We also summarised the adverse effects associated with high-protein intake in studies.

## Materials and methods

This meta-analysis was conducted in accordance with the Preferred Reporting Items for Systematic Reviews and Meta-Analyses (PRISMA) guidelines [23].

### PICOTS

PICOTS (Population [P], Intervention [I], Comparison [C], Outcome [O], Time [T], and Study [S]) was defined before the study search process. Our research question was whether according to RCTs (S), HPDs (I) compared with isocaloric balanced diets (BDs; C) can lead to improved metabolic and reproductive-health outcomes (O) in patients with PCOS (P). Further details regarding the PICOTS criteria are provided in Supplementary Table 1. Because IR and hyperinsulinaemia are major causes of PCOS, we used homoeostatic model assessment for insulin resistance (HOMA-IR) and fasting insulin (FINS) levels as glucose metabolism indicators; total cholesterol (TC), low-density lipoprotein cholesterol (LDL-C), high-density lipoprotein cholesterol (HDL-C), and triglycerides (TGs) as lipid metabolism indicators; and body weight, waist circumference (WC), total testosterone (TT), dehydroepiandrosterone sulphate (DHEAS), sex hormone binding globulin (SHBG), and the free androgen index (FAI) as observation indicators.

### Databases and search strategy

Seven electronic databases, namely MEDLINE (PubMed), Embase, ClinicalTrials.gov, Web of Science, China National Knowledge Infrastructure, Wanfang Data, and Cochrane Library, were searched from inception to 30 April 2023 to identify studies comparing the effects of HPDs and other diets on the IR, anthropometrics, lipid profiles, glucoregulatory outcomes, and hormonal profiles of women with PCOS. The following PCOS-related search terms were used: polycystic ovary syndrome OR polycystic ovar* OR poly-cystic ovar* OR PCOS OR PCO* OR leventhal OR anovulation OR anovulat* OR oligo-ovulat* OR oligoovulat* OR sclerocystic ovary syndrome. In addition, the following diet-related keywords were used: high protein OR high-protein low-carbohydrate OR high protein intake OR carbohydrate-restricted OR diet composition OR high protein. Further details regarding the search strategy are provided in Supplementary Table 2. Reference lists and conference proceedings were manually examined to obtain additional relevant data. The language was restricted to English and Chinese.

### Study selection

The following types of studies were included: (1) studies focusing on women with PCOS; (2) studies evaluating the effects of HPDs and other isocaloric diets, with the proportion of energy supplied by protein representing at least 25% of the total dietary energy intake; (3) parallel or cross-over RCTs; (4) studies with an intervention duration of 4 weeks or more; and (5) studies in which the outcomes included at least one of the following: weight, body mass index (BMI), WC, waist-to-hip ratio (WHR), FINS, HOMA-IR, reproductive hormones, and lipid profile. The following types of studies were excluded: (1) cohort or case–control studies, reviews, meta-analyses, case reports, and animal or cell experiments; (2) studies focusing on pregnancy or lactation; (3) studies involving women with other causes for hyperandrogenism and abnormal ovulation or any serious medical, psychiatric, or neurological problems; and (4) studies with missing data.

### Data extraction

Literature screening was independently conducted by two investigators (FW and PJL). All discrepancies and disagreements regarding study inclusion and exclusion were resolved by consensus or consultation with a third investigator (WW). Basic data were collected using a predesigned data extraction form and included the general characteristics of each study, that is, the (1) name of the first author, year of publication, and country of study; (2) participant characteristics, including the total sample size and actual sample size, age at baseline, duration of intervention, and criteria used to define PCOS; (3) study design and duration of intervention; (4) dietary characteristics, including type, energy, macronutrient composition, ratio, and specific forms of dietary control; and (5) baseline and postintervention metabolic and reproductive outcomes, including weight, BMI, fasting plasma glucose (FPG), glycosylated haemoglobin, TG, LDL-C, TC, HDL-C, HOMA-IR, FINS, TT, DHEAS, SHBG, and the FAI. The completion rates, adverse events, and whether any other interventions were implemented were recorded and assessed. Data were extracted by PJL and examined by FW for any potential errors.

### Quality assessment

Methodological quality was independently evaluated by two researchers (FW and PJL) by using the Cochrane Collaboration tool [24]. All disagreements were resolved through consultation with a third researcher (WW). Studies were evaluated as having low or high bias or unclear risk on the basis of the following: sequence generation, allocation concealment, participant blinding, personnel and outcome assessors, outcome assessment blinding, incomplete outcome data, selective outcome reporting, and other types of bias.

### Data analysis

All statistical analyses were conducted using STATA 14.0 (StataCorp, College Station, TX, USA). Changes in each outcome were reported as differences between mean values before and after the intervention. If the means and standard deviations (SDs) of changes from baseline were specified in the papers, they were directly used. If not, mean changes in the observed parameters were calculated by subtracting the baseline values from the postintervention values. The SD of each difference was calculated as follows:

$${SD}=\sqrt{{\rm{SD}}1* {\rm{SD}}1+{\rm{SD}}2* {\rm{SD}}2-2{\rm{R}}* {\rm{SD}}1* {\rm{SD}}2}\,({\rm{R}}=0.5)$$

If the target data in an included study were expressed as medians (quantile interval), the mean value of the target data was determined using the method of Luo et al. [25] or Hozo et al. [26] when appropriate. In brief, if the sample size in an included study is >25, then the median is considered as the mean value of target data; while the sample size in an included study is less than or equal to 25, the estimated average value is calculated using the following formula based on the data format given in the included study:

Mean = (a + 2*m + b)/4 OR Mean = (q1 + m + q3)/3 [a, b, and m represent the minimum, maximum, and median values of the target data, respectively; q1 or q3 represents the cut-off value of the first or third quantile of the target data, respectively].

In addition, depending on the characteristics of the included data, the SDs of the target data were calculated using the method of Wan et al. [27] or Hozo et al. [26]. In short, the estimated SDs of the target data were calculated as (*b* − *a*) / 4 [the sample size of the included study is <70] or (*b* − *a*) / 6 [the sample size of the included study is of ≥70] {a and b represent the minimum, maximum values of the target data, respectively}

Subsequently, the results were pooled for meta-analysis as mean differences with 95% confidence intervals (CIs). Statistical significance was set at *P* < 0.05. The heterogeneity within comparisons was evaluated using Cochran’s Q test and quantified using the *I*^2^ statistic. *I*^2^ values of <25%, 25%–50%, and >50% represented low, moderate, and high heterogeneity, respectively [28].

A random-effects method was used to calculate summary effect measures at *I*^2^ > 0%. Sensitivity analysis was then conducted by including only studies with a low risk of bias. Subgroup analysis was conducted in cases involving more than three studies. The subgroups were categorised on the basis of the intervention duration (<12 and ≥12 weeks) and ethnicity. When required, Egger’s test and funnel plots were used to investigate potential publication bias [29, 30].

## Results

### Study characteristics

**Fig. 1:**
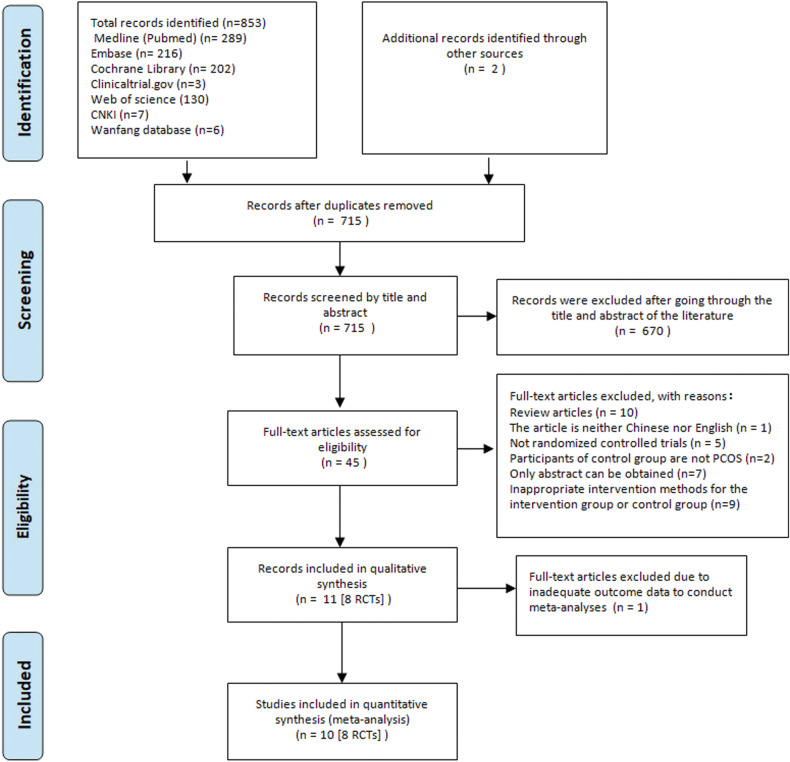
Literature screening process.

In the preliminary search, 853 studies were identified. We identified 10 articles reporting on 8 RCTs that involved a total of 300 participants (154 in the intervention group and 146 in the control group) as eligible for meta-analysis [31–40]. Further details regarding the selection process are provided in the PRISMA flow diagram in Fig. 1.

Table 1 presents the general characteristics of the included studies, which were published between 2003 and 2021. These studies were parallel-design, single-centre trials conducted in the United States [32, 37], Australia [33, 34], Brazil [31], Iran [35, 36, 38], and China [38, 39]. All studies included women with overweight and obesity (BMI ≥ 24 kg/m^2^), except one [31] recruited patients without BMI limitations. The participants of the Toscani study [31] were aged between 14–35, whereas those in other studies were between 18–45 years old. All participants received a diagnosis of PCOS based on the criteria of the National Institutes of Health (NIH) [32–35, 37], Rotterdam consensus [36–38], or Androgen Excess and Polycystic Ovary Syndrome (AE-PCOS) [31], with one participant not receiving a diagnosis [40]. The intervention duration ranged from 4–16 weeks. All studies focused on HPDs as an exposure factor and compared the effects of HPDs and isocaloric BDs. Three studies used protein powder as a protein administration method for the HPD intervention group [36, 38, 39], whereas the other studies used a high-protein dietary pattern as an intervention measure.

**Table 1:** Summary of the studies included in our analysis.

Author, year (reference), Country	Participants, characteristics, and PCOS definitions	RCT designs (blinding)	Duration	Intervention diet characteristics	Control diet characteristics	Main outcomes measures in analysis
Toscani et al., 2011 [31], Brazil	Total completers, 18 (Case, 9; control, 9); age (case/control): (22.72±5.68)/(29.35±5.74); BMI: 18.5-39.9; PCOS definition: AE-PCOS	Parallel (yes, only participants)	8 weeks	A HP diet: P, 30%; F, 30%, CHO, 40%; 20–25 kcal/kg current weight/day for participants and 25–30 kcal/kg current weight/day for normoweight participants) Diet	Control diet: P, 15%; F, 30%; CHO, 55%; 20–25 kcal/kg current weight/day for participants and 25–30 kcal/kg current weight/day for normoweight women)	Weight, WC, fasting glucose, fasting insulin, HOMA, TC, TG, HDL-c, LDL-c
Stamets et al., 2004 [32], USA	Total completers, 26 (Case, 13; control, 13); Age: 21-37 years; BMI(case/control): (38±4)/(37±4); PCOS definition: NIH	Parallel (NR)	1 month	A hypocaloric HP diet: P, 30%; F, 30%, CHO, 40%; Diet	A hypocaloric high-carbohydrate diet: P, 15%; F, 30%; CHO, 55%;	Weight, WC, WHR, TT, DHEAS, TC, TG, HDL-c, LDL-c
Moran et al., 2010 [33], 2003 [34], Australia	Total completers, 28 (Case, 14; control, 14); age (case/control): (33±1.2)/(32±1.2); BMI(case/control): (37.9±1.6)/(37.7±1.9); PCOS definition: NIH	Parallel (Participants and investigators were not blinded)	16 weeks	An energy-restricted diet (wk 0-12), following a weight maintenance diet for 4 weeks; HP diet: P, 30%; F, 30%, CHO, 40% Diet	An energy-restricted diet (wk 0-12), following a weight maintenance diet for 4 weeks; Control diet: P, 15%; F, 30%; CHO, 55%	Weight, BMI, fasting glucose, fasting insulin, HOMA, TC, TG, HDL-c, LDL-c, FAI
Mehrabani et al., 2012 [35],Iran	Total completers, 49 (Case, 23; control, 26); age: 20-40; BMI: 25-38; PCOS definition: NIH	Parallel (NR)	12 weeks	A low-GL and HP diet (P, 30%; F, 30%, CHO, 40%) Diet	A conventional hypocaloric diet (P, 15%; F, 30%; CHO, 55%)	Weight, HOMA, TC, TG, HDL-c, LDL-c TT, SHBG, FAI, DHEAS, fasting insulin, HOMA-IR
Nadjarzadeh et al., 2021 [38]; Elham HM, et al., 2020 [36], Iran	Total completers (groups without fennel supplementation), 30 (Case, 15; control, 15); age: 18–45; BMI: ≥ 25; PCOS definition: Rotterdam criteria	Parallel (yes, participants and investigators)	3 months	A hypocaloric HP, low-carbohydrate diet (P, 30%; F, 30%, CHO, 40%) Diet	A hypocaloric standardized diet: P, 15%; F, 30%; CHO, 55%	Weight, BMI, WC, WHR, TT, SHBG, FAI, fasting glucose, fasting insulin, HOMA-IR,
Kasim-Karakas et al., 2009 [37], USA	Total completers, 24 (Case, 13; control, 11); Age: 18–45 years; BMI(case/control): (38.9±1.6)/(35.4±1.8); PCOS definition: NIH	Parallel (yes, only participants)	2 months	An energy-restricted HP diet: P, 33.7%; F, 26.2%, CHO, 39.5% Whey	An energy-restricted control diet: P, 16.6%; F, 25.9%; CHO, 56.7%	Weight, BMI, fasting glucose, fasting insulin, HOMA, TC, TG, HDL-c, TT, SHBG, FAI, DHEAS
Dou et al., 2023 [39], China	Total completers, 52 (high protein arm, 30; control, 22); age: 22–44 years; BMI: 28.07±3.59; PCOS definition: Rotterdam criteria	Parallel (non-blind)	8 weeks	HP diet: P, 1.5–2.0g/kg/d; F and CHO, NR Whey	An energy-restricted diet (P, 10-20%, F, 25–30%; CHO, 55-60%)	Weight, BMI, fasting glucose, fating insulin, HOMA-IR, TT, SHBG, FAI
Chen et al., 2021 [40], China	Total completers, 73 (Case, 37; control, 36); age: 18–40 years; BMI: ≥28; PCOS definition: NR	Parallel (NR)	3 months	An energy-restricted HP diet (P, 30%; F, 30%, CHO, 40%) Whey	An energy-restricted diet (the composition of macronutrients is not reported)	BMI, WHR, TC, TG, HDL-c, fasting glucose, fating insulin

*AE-PCOS* Androgen Excess and Polycystic Ovary Syndrome, *BMI* body mass index, *CHO* carbohydrate, *DHEAS* dehydroepiandrosterone sulphate, *F* fat, *FAI* free androgen index, *GL* glycaemic load, *HDL-C* high-density lipoprotein cholesterol, *HOMA-IR* homoeostatic model assessment for insulin resistance, *HP* high protein, *LDL-C* low-density lipoprotein cholesterol, *NR* not reported, *PCOS* polycystic ovary syndrome, *RCT* randomised controlled trial, *SHBG* sex hormone binding globulin, *TC* total cholesterol, *TG* triglyceride, *TT* total testosterone, *WC* waist circumference, *WHR* waist-to-hip ratio.

### Meta-analysis results

#### Anthropometrics

##### Weight

**Fig. 2:**
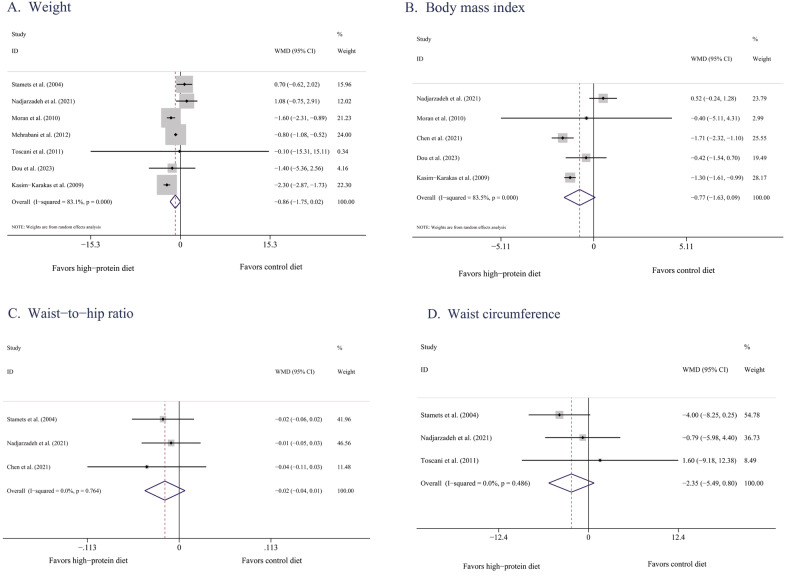
Effects of HPDs on anthropometrics in women with PCOS. **A** Weight**/**Forest plots of the effect of HPDs on weight. **B** Body mass index**/**Forest plots of the effect of HPDs on BMI. **C** Waist-to-hip ration/Forest plots of the effect of HPDs on WHR. **D** Waist circumference**/**Forest plots of the effect of HPDs on WC.

According to data pooled from seven of the eligible studies [31–33, 35, 37–39], HPDs did not significantly reduce body weight when compared with BDs (−0.78 kg, 95% CI [−1.69, 0.13], *P* = 0.06; Fig. 2A). The degree of heterogeneity was high (*I*^2^ = 82%). Removing any of the seven studies did not cause the *I*^2^ to decrease to below 50%.

When conducting subgroup analysis by country, after a Chinese study was excluded [39], no significant changes were observed in the results (−0.83 kg, 95% CI [−1.75, 0.09], *P* = 0.08, *I*^2^ = 86%). According to the subgroup analysis results for intervention duration, HPDs were not superior to BDs in terms of leading to weight loss when they were implemented as short-term (<12 weeks) [31, 32, 37, 39] or long-term (≥12 weeks) [33, 35, 38] interventions (*P* = 0.42 and 0.09, respectively), with high heterogeneity noted in the subgroups (82% and 77%). In addition, according to the subgroup analysis results for ethnicity, HPDs and BDs had similar effects (*P* = 0.07 and 0.65, respectively; Supplementary Table 3).

##### BMI

According to data pooled from five eligible studies [33, 37–40], when compared with BDs, HPDs did not significantly reduce BMI (−0.81 kg/m^2^, 95% CI [−1.69, 0.07], *P* = 0.07; Fig. 2B). The degree of heterogeneity was high (*I*^2^ = 83%). After the study by Nadjarzadeh et al. [38] was excluded, sensitivity analysis revealed a change in the overall effect size (−1.36, 95% CI [−1.63, −1.08], *P* < 0.0001, *I*^2^ = 0).

After the two Chinese studies [39, 40] were excluded, no significant changes were observed in the results (−0.42 kg/m^2^, 95% CI [−2.06, 1.21], *P* = 0.61, *I*^2^ = 89%). In terms of intervention duration, HPDs did not significantly reduce BMI when implemented as long-term interventions (−0.58 kg/m^2^, 95% CI [−2.55, 1.38], *P* = 0.56, *I*^2^ = 90%) [33, 38, 40]. According to the subgroup analysis results for ethnicity, HPDs and BDs had similar effects on Asian populations (*P* = 0.47, Supplementary Table 3).

##### Abdominal obesity

Three studies [32, 38, 40] reported a change in WHR (−0.02, 95% CI [−0.04, 0.01], *P* = 0.16, *I*^2^ = 0%; Fig. 2C). Similarly, three studies [31, 32, 38] reported a change in WC (−2.56 cm, 95% CI [−5.91, 0.79], *P* = 0.13, *I*^2^ = 0%; Fig. 2D). These results indicate that both interventions similarly reduced WC and WHR. However, because the number of studies reporting such data was limited, no subgroup analysis was conducted.

#### Glucoregulatory indicators

##### FINS

**Fig. 3:**
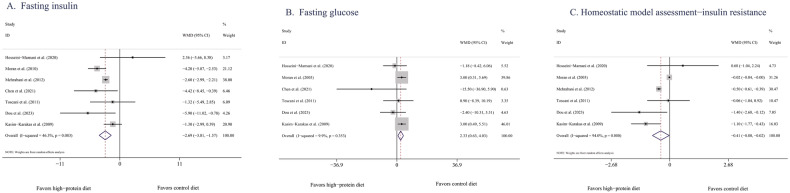
Effects of HPDs on glucoregulatory indicators in women with PCOS. **A** Fasting insulin**/**Forest plots of the effect of HPDs on FINS. **B** Fasting glucose**/**Forest plots of the effect of HPDs on fasting glucose. **C** Homoeostatic model assessment-insulin resistance**/**Forest plots of the effect of HPDs on HOMA-IR.

According to data pooled from seven eligible studies [31, 33, 35–37, 39, 40], when compared with BDs, HPDs significantly reduced concentrations of FINS (−2.69 mIU/mL, 95% CI [−3.81, −1.57], *P* < 0.00001, *I*^2^ = 46%; Fig. 3A). Removing any of these studies did not decrease the *I*^2^ to be <50%. After the two Chinese studies [39, 40] were excluded, no significant changes were observed in the results (−2.41 mIU/mL, 95% CI [−3.6, −1.21], *P* < 0.0001, *I*^2^ = 54%).

In the long-term studies (≥12 weeks) [33, 35, 36, 40], the concentrations of FINS were significantly lower in the HPD group than in the BD group (−3.04 mIU/mL, 95% CI [−4.56, −1.51], *P* < 0.0001, *I*^2^ = 56%). However, in the short-term studies [31, 37, 39], HPDs and BDs had similar effects (−2.58, 95% CI [−5.66, 0.51], *P* = 0.1, *I*^2^ = 55%). In the studies focusing on FINS levels, with the exception of the study by Toscani et al. [31], all participants were women with overweight or obesity. According to the pooled data on the women with PCOS in these studies, the concentration of FINS significantly decreased after HPD interventions (−2.86 mIU/mL, 95% CI [−4.17, −1.54], *P* < 0.0001, *I*^2^ = 59%). The results of the subgroup analysis were consistent between the studies involving Asian populations and studies involving European and American populations, with these groups having effect sizes of −2.88 mIU/mL (95% CI [−5.02, −0.73), *P* = 0.009) and −2.49 mIU/mL (95% CI [−4.73, −0.24], *P* = 0.003), respectively (Supplementary Table 3).

#### FPG

Six studies [31, 34, 36, 37, 39, 40] reported changes in FPG levels before and after the intervention. Pooled analysis revealed a significantly higher concentration of FPG (2.33 mg/dL, 95% CI [0.63, 4.03], *P* = 0.007, *I*^2^ = 10%) in the HPD group than in the BD group (Fig. 3B). After the two Chinese studies [39, 40] were excluded, no significant changes were observed in the results [2.68 mg/dL, 95% CI [0.93, 4.43], *P* = 0.003, *I*^2^ = 0%]. In terms of intervention duration, subgroup analysis revealed that compared with BDs, HPDs were associated with higher concentrations of FPG (3.19 mg/dL, 95% CI [0.97, 5.42], *P* = 0.005, *I*^2^ = 0%) in short-term studies [31, 37, 39]. However, in long-term studies (≥12 weeks) [34, 36, 40], HPDs and BDs had similar effects on the concentrations of FPG [0.39 mg/dL, 95% CI [−5.39, 6.17], *P* = 0.89, *I*^2^ = 47%]. These results indicate that HPD interventions lasting 8 weeks or less may be associated with higher concentrations of FPG than those associated with BD interventions lasting 8 weeks or less. However, for interventions lasting 12 weeks or more, HPDs and BDs have similar effects (Supplementary Table 3).

According to the subgroup analysis results for ethnicity, HPDs significantly increased the concentrations of FPG among European and American populations (2.92 mg/dL, 95% CI [1.12, 4.72], *P* = 0.001, *I*^2^ = 0%) but not among Asian populations (−2.54 mg/dL, 95% CI [−7.72, 2.64], *P* = 0.34, *I*^2^ = 0%).

##### HOMA-IR

According to data pooled from six eligible studies [31, 33–39], HPDs and BDs had different effects on HOMA-IR (−0.41, 95% CI [−0.78, −0.03], *P* = 0.03; Fig. 3C). The degree of heterogeneity was high (*I*^2^ = 96%). After the two studies by Moran et al. [33, 34] were excluded (*I*^2^ = 39%), no significant changes were observed in the results (−0.58, 95% CI [−0.95, −0.20], *P* = 0.003).

In the long-term studies [33–36, 38], HPDs and BDs had similar effects on HOMA-IR (−0.20, 95% CI [−0.65, 0.25], *P* = 0.38, *I*^2^ = 97%). However, in the short-term studies [31, 37, 39], HPDs resulted in a significantly larger reduction in HOMA-IR (−0.82, 95% CI [−1.29, −0.35], *P* = 0.0006, *I*^2^ = 35%; Supplementary Table 3).

#### Blood lipid profiles

##### TC

**Fig. 4:**
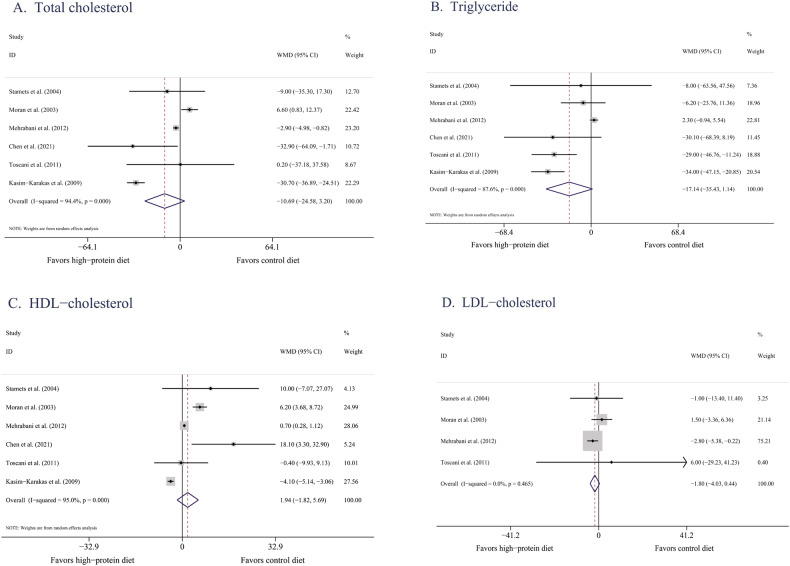
Effects of HPDs on blood lipid profiles in women with PCOS. **A** Total cholesterol/Forest plots of the effect of HPDs on TC. **B** Triglyceride/Forest plots of the effect of HPDs on TGs. **C** High density lipoprotein cholesterol**/**Forest plots of the effect of HPDs on HDL-C. **D** low density lipoprotein cholesterol/Forest plots of the effect of HPDs on LDL-C.

Six studies [31, 32, 34, 35, 37, 40] reported changes in TC. Meta-analysis revealed no significant difference between the changes in TC resulting from HPDs and BDs in these studies (−10.68 mg/dL, 95% CI [−24.57, 3.21], *P* = 0.13, *I*^2^ = 94%; Fig. 4A). Subgroup analysis revealed no significant difference in the effects of HPDs and BDs on TC in either short-term (<12 weeks) or long-term (≥12 weeks) interventions (*P* = 0.06 and 0.79, respectively; Supplementary Table 3). Removing any of these studies did not cause the *I*^2^ to decrease to below 50%.

##### TG levels

Six studies [31, 32, 34, 35, 37, 40] reported changes in TG levels. Pooled analysis revealed no significant difference between the influence of HPDs and BDs on these levels (−17.13 mg/dL, 95% CI [−35.37, 1.11], *P* = 0.07, *I*^2^ = 88%; Fig. 4B). After a Chinese study [40] was excluded, no significant changes were observed in the results (*P* = 0.12). In the short-term studies (4–8 weeks) [31, 32, 37], HPDs were significantly more effective than BDs were in reducing TG levels (−31.38 mg/dL, 95% CI [−41.77, −21.00], *P* < 0.0001, *I*^2^ = 0%). However, in the long-term studies (≥12 weeks) [34, 35, 40], no significant difference was observed in the effects of HPDs and BDs on TG reduction (Supplementary Table 3). Even when the studies were removed one by one, the degree of heterogeneity remained high.

##### HDL-C

Six studies [31, 32, 34, 35, 37, 40] reported changes in HDL-C. Their results revealed no significant difference between the influence of HPDs and BDs on HDL-C (1.94 mg/dL, 95% CI [−1.82, 5.69], *P* = 0.31, *I*^2^ = 95%; Fig. 4C). According to the subgroup analysis results for intervention duration, similar outcomes were observed in the short- and long-term studies (*P* = 0.46 and 0.08, respectively; Supplementary Table 3). Even when studies were removed one by one, the degree of heterogeneity remained high.

##### LDL-C

Four studies [31, 32, 34, 35] reported changes in LDL-C. Their results revealed no significant difference between the influence of HPDs and BDs on LDL-C (−1.80 mg/dL, 95% CI [−4.03, 0.44], *P* = 0.11, *I*^2^ = 0%; Fig. 4D). Because the number of studies reporting on LDL-C was limited, no subgroup analysis was conducted.

#### Reproductive hormones

##### TT

**Fig. 5:**
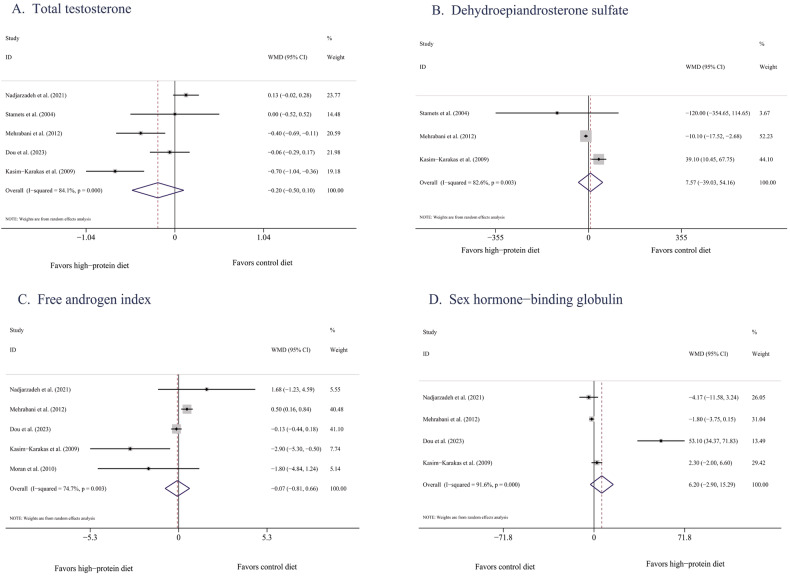
Effects of HPDs on reproductive hormones in women with PCOS. **A** Total testosterone/Forest plots of the effect of HPDs on TT. **B** Dehydroepiandrosterone**/**Forest plots of the effect of HPDs on DHEAS. **C** Free androgen index/Forest plots of the effect of HPDs on the FAI. **D** Sex hormone-binding globulin**/**Forest plots of the effect of HPDs on SHBG.

According to data pooled from five eligible studies [32, 35, 37–39], HPDs did not significantly reduce concentrations of TT (−0.20 nmol/L, 95% CI [−0.50, 0.10], *P* = 0.20) compared with isocaloric BDs (Fig. 5A). The degree of heterogeneity was high (*I*^2^ = 84%, *P* < 0.0001). After the study by Nadjarzadeh et al. [38] was excluded, the *I*^2^ decreased to 47%, with an effect size of −0.39 nmol/L (95% CI [−0.71, −0.07], *P* = 0.02). Subgroup analysis revealed no significant difference in the effects of HPDs and isocaloric BDs on the concentrations of TT, regardless of intervention duration and ethnicity (Supplementary Table 3).

##### DHEAS

According to data pooled from three eligible studies [32, 35, 37], HPDs did not significantly reduce concentrations of DHEAS (7.67 ng/mL, 95% CI [−38.78, 54.11], *P* = 0.75, *I*^2^ = 87%) compared with isocaloric BDs (Fig. 5B).

##### FAI

According to data pooled from five eligible studies [33, 35, 37–39], no significant differences were observed in the effects of HPDs and isocaloric BDs on the FAI (−0.07, 95% CI [−0.81, 0.66], *P* = 0.84, *I*^2^ = 75%; Fig. 5C). According to the subgroup analysis results for three long-term studies [33, 35, 38], HPDs and isocaloric BDs had similar effects on the FAI (0.37, 95% CI [−0.84, 1.57], *P* = 0.55, *I*^2^ = 29%). Additionally, according to the combined results of three studies [35, 38, 39], HPDs and BDs had similar effects on the Asian populations (*P* = 0.24, Supplementary Table 3).

##### SHBG

Four studies reported changes in the concentrations of SHBG [35, 37–39]. Pooled analysis revealed that neither HPDs nor isocaloric BDs significantly reduced the concentrations of SHBG after the intervention (6.20 nmol/L, 95% CI [−2.90, 15.29], *P* = 0.18, *I*^2^ = 92%; Fig. 5D). However, because the number of studies reporting concentrations of SHBG was limited, no subgroup analysis was conducted.

### Study quality and risk of bias

**Fig. 6:**
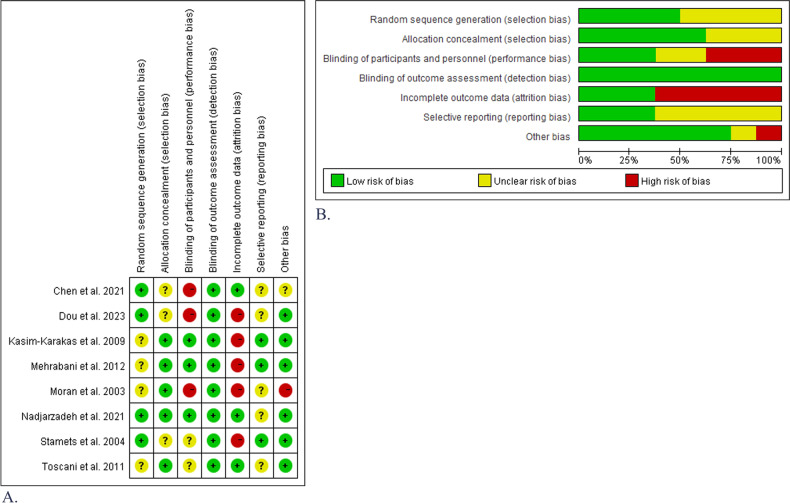
Risk of bias assessment. **A** Risk of bias in seven domains for all included studies. **B** Risk of bias assessment across seven domains for each included study.

A quality assessment of each trial is presented in Fig. 6A and B, and the details are provided in Supplementary Table 4. Four studies [32, 38–40] provided detailed information regarding their randomisation methods, and five studies [31, 33, 35, 37, 38] explained their methods of allocation concealment. The risk of intervention blinding was high in three studies [34, 39, 40], low in three studies [35, 37, 38], and unclear in two studies [31, 32]. Data analysis blinding was performed in all studies. Five studies [32, 34, 35, 37, 39] had a high risk of reporting bias, and three studies [31, 38, 40] had a low risk of reporting bias.

## Discussion

Diet control is an essential technique for achieving or maintaining the optimal weight for women with PCOS, although no consensus has yet been reached regarding the optimal distribution of dietary components for such women [19]. Multiple studies have indicated that HPDs have positive effects on obesity, metabolic syndrome, and diabetes [16–18]. However, no systematic meta-analysis has specifically summarised the effects of HPDs on PCOS. We reviewed eight RCTs involving a total of 300 women. Our results indicate that, compared with BDs, HPDs are associated with a significantly greater reduction in FINS and HOMA-IR levels in patients with PCOS. However, HPDs and BDs have similar effects on weight, abdominal obesity, lipid profiles, and sex hormone levels.

Our finding, that HPDs and BDs with the same energy restrictions have similar effects on body weight, BMI, and abdominal obesity, is consistent with the previous studies with similar intervention length [34, 41–43]. Collectively, it appears that when energy intake is controlled, the distribution of macronutrients does not affect the amount of weight loss. In the current study, we noted a high degree of heterogeneity in the pooled results of weight and BMI among the included studies, which can be explained by differences in the durations of interventions and the ethnicities. Some studies have indicated extending HPD interventions (e.g., 4 weeks or more) may promote the loss of body weight and fat and improve subsequent weight maintenance [41, 44]. Our subgroup analysis revealed a long-term intervention of 12 weeks or more increased the trend of weight loss, although the results did not reach statistical significance. The mechanism underlying the effects of HPDs on weight loss is likely linked to high energy expenditure. Compared to BDs, HPDs could maintain or increase lean body mass [45], which is a predominant contributor to resting energy expenditure (REE). The HPDs were found to bring less REE reduction during weight loss whereas others did not [46]. Moreover, protein consumption has higher dietary thermogenesis than carbohydrates or fat due to different nutrient processing [47]. HPDs also contribute to a greater satiating effect and the following reduction in food consumption and weight [48]. One of the explanations for such satiety is the increased levels of anorexigenic hormones, such as glucagon-like peptide-1, cholecystokinin, and peptide tyrosine-tyrosine [49, 50]. These hormones and vagal afferent fibres can work on brain regions related to reward and motivation, hypothalamus, and other regions responsible for energy homoeostasis, and regulate people’s dietary consumption and adherence to weight control programmes [51]. Our results for weight and BMI showed the tendency for HPD to lead to more reduction of weight and BMI (*P* = 0.06 and 0.07), however, since total energy intake prescribed in HP and control groups were comparable and low in most studies, the difference was not significant. It may also be possible that a 4–12 weeks intervention is not long enough to see the difference in adherence or the effect of satiety from HPD on total energy intake. The lower spontaneous energy intake led by satiating effect and adherence of HPD might be seen when there is free dietary choice rather than isoenergetic with the comparative diet. Although no changes in fat mass were reported in the included studies, they used WC and WHR to assess the abdominal fat improvement, and the results revealed that HPDs did not significantly reduce abdominal obesity. Therefore, current evidence indicates that different diets with isoenergetic restriction can promote similar weight loss and the calorie deficit is fundamental for weight control.

In terms of IR, we discovered HPDs were more effective than BDs in improving the levels of FINS and HOMA-IR in patients with PCOS. Subgroup analysis revealed that HPDs and isoenergetic BDs had a similar effect on the concentration of FINS in short-term interventions (8 weeks or less) whereas that HPDs offer additional advantages in long-term interventions (12 weeks or more). Subgroup analysis of ethnicity also revealed an advantage of HPDs in terms of FINS reduction both in European, American, and Asian populations. Our findings are consistent with those who reported that HPD interventions improved the FINS and insulin homoeostasis [18, 42, 52]. Protein and amino acid intake are known to enhance insulin secretion, which is associated with a more than compensatory increase in insulin clearance, thus resulting in lower plasma insulin levels [53, 54]. Moreover, HPDs is with a corresponding reduction of carbohydrates, which could improve insulin sensitivity, enhance pancreatic β-cell function and endogenous insulin clearance [55]. Some studies conducted among participants with overweight or obesity observed a larger reduction of FINS with HPD than average-protein diets, although there were similar effects on weight and FPG change [56]. Our analysis revealed a mild reduction in HOMA-IR, with this reduction primarily attributable to the findings of Dou et al. [39]. When this study was excluded, pooled analysis revealed the two interventions had similar effects on HOMA-IR. Because HOMA-IR here was calculated using a formula instead of a hyperinsulinaemic (euglycaemic) clamp [57], the value was influenced by both FPG and FINS. According to a previous study [58], insulin levels change more rapidly than FPG levels do, which may explain the substantial reductions observed in FINS and HOMA-IR in the current analysis. Overall, these findings indicate that compared with BDs, HPDs are associated with considerably more favourable insulin and HOMA-IR.

We investigated the effects of HPDs on the metabolism of glucolipids and observed a slight increase in FPG levels when HPDs were implemented (2.33 mg/dL, 95% CI [0.63, 4.03], *P* = 0.007, *I*^2^ = 10%). As an indicator of instantaneous blood glucose, FPG is influenced by various factors and cannot be solely relied upon as an indicator of blood glucose control. FPG findings are also typically influenced by the level of glucose at baseline, the source of protein, and the study duration. The effect of HPD on lipids is mostly through weight loss. Although the short-term studies analyzed in the current meta-analysis revealed a favourable effect of HPDs on TG reduction, this advantage was not observed in the long-term studies, which is presumably because of the comparable energy balance and fat content between the groups, so as the similar weight and fat reduction in these studies.

We noted both a decrease in the FAI and levels of TT and an increase in the levels of SHBG and DHEAS in the patients with PCOS, regardless of their dietary patterns. These findings are consistent with those of one previous study indicating that even modest weight loss of 5%–10% over a short duration of 4 weeks may lead to improvements in PCOS symptoms [32], including those participants who still had obesity or overweight. Thus, regardless of whether a high-protein model is adopted, energy restriction is essential for achieving hormonal improvements. However, HPDs did not show an advantage in modulating endocrine hormones compared to BDs, which may have resulted from similar weight loss or the short duration of the intervention.

During the data merging process, we encountered a high degree of heterogeneity. To explore the potential effects of such heterogeneity on our results, we conducted several subgroup analyses involving factors such as baseline BMI category, ethnicity, and intervention duration. Nevertheless, heterogeneity persisted in the data, presumably because of the inherent complexity of patients with PCOS, who exhibit different disease phenotypes and may require additional clinical interventions alongside dietary modifications. Inadequate reporting of medication usage also contributed to the heterogeneity in the included studies.

Some researchers raised concerns about the potential risks following HPD, such as osteoporosis and kidney damage, however none of the studies included in our analysis reported any serious adverse events associated with HPDs, thus high-protein intake which provides around 30% of dietary calories or 1.5–2.0 g/kg/d is generally safe in women with PCOS. Theoretically, HPD may promote urinary calcium excretion resulting in calcium loss, but protein also increases intestinal calcium absorption and circulating insulin-like growth factor-I, while decreasing parathyroid hormone, which sufficiently counteracts the negative effects of protein acid loading on bone health. Therefore, systemic calcium homoeostasis and bone status were not negatively affected by the increased acid load associated with high protein [59], and the previous evidence did not identify a significantly unfavourable effect of protein intake on lumbar spine bone mineral density [60]. Due to the increased urinary calcium loss, nephrolithiasis is another risk of HPD intervention. However, with weight loss, the risk of metabolic syndrome-associated nephrolithiasis could be reduced [61]. Overall, it remains prudent to check with medical history and perform the necessary tests to assess the risk of nephrolithiasis before starting an HPD regimen.

Renal function is also an aspect that should be checked before HPD intervention because high protein consumption may increase glomerular hyperfiltration, but there is little evidence for such side effects within 24 months of HPD intervention in people without established renal disease [16, 62, 63]. In turn, weight loss reduces obesity-related kidney damage by decreasing renal filtration rate. However, HPD has the potential to cause further decline in renal function in patients with renal insufficiency and should be avoided [63, 64]. Because chronic kidney disease is often silent, patients should be screened for kidney function, such as serum creatinine and proteinuria, before the initiation of HP intervention. The upper limit of protein intake and intervention duration is not clearly defined, but based on included studies, basically, HPD up to 1.66 g/kg/day or accounts for 25–30% of the total energy intake for 4–16 weeks does not pose adverse effects.

To evaluate potential bias in favour of intervention adherence, we calculated the completion rates of the included studies. In the trials lasting 12 weeks or more [33–36, 38, 40], 91.6% of the patients completed the intervention, whereas in the trials lasting <12 weeks, 81% of the patients completed the intervention. No significant differences were observed between the intervention and control groups, indicating a high level of compliance with the dietary approach. However, the included studies did not describe specific information about the source and quality of protein, fats, and carbohydrates, especially the glycemic index, which are potential factors affecting metabolism. Moreover, exercise compliance may also have an impact on the results and should be rigorously standardised and reported, analysed as potential confounders in the future studies, in order to make the results more directly applicable to the clinical workplace.

To our knowledge, this is the first meta-analysis to summarise the effects of HPDs on women with PCOS. The strength of this study is that it included RCTs involving patients with PCOS undergoing high-protein interventions and relative subgroup analyses as a means of reducing study heterogeneity. However, some of the included studies did not report the SDs of change from baseline, which is one of the limitations. Therefore, the data were handled using Follman’s formula, and the results may have been affected using calculation. The second limitation is that the limited duration of intervention (4–16 weeks) in current studies, covering up the truly long-term effects of HPDs and calling for the requirement of further research focusing longer intervention. More importantly, when conducting subgroup analysis, we found that different intervention duration may be associated with different metabolic improvement, so future research could have a longer intervention duration, and monitor the metabolic changes at different time points in order to understand the metabolic improvement effects of different intervention duration. Furthermore, heterogeneity was found in many comparisons, rendering the difficulty in utility in clinical practice, even though we tried out best to minimize it by performing subgroup analysis. In addition, the number of people included in this meta-analysis is limited, with participants of each group in the studies ranging from 9–37. Thus, the multi-centre with larger sample size studies are needed in the further research in order to make the results more reliable. The final limitation is the ratio of carbohydrate in the control group was higher than that in intervention diet, therefore it is difficult to determine which nutrient affects the outcomes of interest. It is the fact that, high protein is along with low in carbohydrate when fat intake fixed in a food pattern, but we could do more basic research on the metabolic pathway to identify whether the improvement is contributed to high protein or low carbohydrate, or both.

## Conclusion

Compared with isoenergetic BDs, HPDs are associated with more favourable improvements in FINS and HOMA-IR, whereas they have similar effects on body weight, abdominal obesity, lipid metabolism, and sex hormones. Therefore on the basis of limiting the total energy, HPDs could be adopted as nutritional intervention for PCOS management in clinical practice, especially for insulin resistance improvement, but it is significant to screen kidney function and risk of nephrolithiasis before HPD intervention. Due to the high heterogeneity, further research with consideration of different phenotypes of PCOS in larger and broader settings and longer intervention duration is required. In addition, the overall effect of HPDs and potential mechanism behind this should be further elucidated beyond the context of intermediate biomarkers and other crucial clinical outcomes, such as the risks of diabetes, cardiovascular disease, ovulatory cycle disturbances, and infertility in patients with PCOS.

### Supplementary information

SUPPLEMENTARY MATERIAL
