# Supplementary material for: Effects of high-protein diets on the cardiometabolic factors and reproductive hormones of women with polycystic ovary syndrome: a systematic review and meta-analysis
Source: Nutr Diabetes. 2024 Feb 29;14:6. doi: 10.1038/s41387-024-00263-9 (PMC10904368; doi:10.1038/s41387-024-00263-9)
Supplement: Supplementary file 1 — SUPPLEMENTARY MATERIAL [file 41387_2024_263_MOESM1_ESM.pdf]

SUPPLEMENTARY MATERIAL

Contents

Supplementary Table 1. Summary of the PICO criteria used to identify studies to be included

Supplementary Table 2. Search strategy of the systematic review

Supplementary Table 3. Subgroup analyses

Supplementary Table 4. Risk of bias assessment in each study

Supplementary Table 1. Summary of the PICTOS criteria used to identify studies to be included	
Parameter	Description
Population	Adult women with PCOS (diagnosed by the NIH 1990 (**a**), Rotterdam 2003 (**b**), or AEPCOS 2006 criteria (**c**))
Intervention	A high-protein diet (Energy from protein was not less than 25% of total dietary energy intake)
Comparison	The comparison group of the same study with control diet (a normal-protein diet or conventional calorie-restricted diet, with calorie from protein is less than 25% of total dietary energy intake)
Outcome	Cardiometabolic and reproductive outcomes reported at the beginning and the end of the trial for both intervention and control groups as described below:<br>Outcomes: HOMA-IR as a marker of insulin resistance, or cardiometabolic profile (fasting insulin; fasting glucose); lipid profile (TC, LDL-C, HDL-C, TG); body weight and WC; and, (2) reproductive profile (TT, FAI, SHBG, DHEAS)
Time	The duration of the study was not less than 4 weeks
Study design	Randomized controlled trial (parallel or cross-over designs)

Abbreviations: PICOTS, population – intervention – comparison – outcome – time – study design; PCOS, polycystic ovary syndrome; NIH, National Institutes of Health, AEPCOS, Androgen Excess and PCOS Society; RCT, randomized controlled trial; HOMA-IR, homeostatic model assessment of insulin resistance; TC, total cholesterol; LDL-C, low-density lipoprotein cholesterol; HDL-C, high-density lipoprotein cholesterol; TG, triglyceride; WC, waist circumference; TT, total testosterone; FAI, free androgen index.

Supplementary Table 2. Search strategy of the systematic review

PCOS key terms		Diet key terms		Limitations	
1)	polycystic ovary syndrome/	12)	exp high protein/	19)	11 AND 18
2)	polycystic ovar*.mp.	13)	exp high-protein low-carbohydrate/		
3)	poly-cystic ovar*.mp.	14)	exp high protein intake/		
4)	PCOS or PCO*.mp.	15)	exp Carbohydrate-Restricted/		
5)	leventhal.mp.	16)	exp diet composition/		
6)	anovulation/	17)	high protein.mp.		
7)	anovulat*.mp.	18)	OR/ 12-17		
8)	oligo-ovulat*.mp.				
9)	oligoovulat*.mp.				
10)	sclerocystic ovary syndrome.mp.				
11)	OR/ 1-10				

Abbreviations/symbols: **/**, Medical Subject Heading (MeSH) for MEDLINE; *, any character; mp., multipurpose (searches several fields including the MEDLINE title, original title, abstract, subject heading, name of substance, and registry word fields).

The present search strategy was developed for MEDLINE and was modified as appropriate for other databases.

Original search was conducted in April 30, 2023.

Supplementary Table 3. Subgroup analyses for the effects of a high-protein diet on profiles of anthropometric indices, glucoregulatory status, lipids, and hormone status in women with polycystic ovary syndrome.

Subgroup by	No. of trials	Effect size	95% CI	P for effect estimates	I^2, %	P for heterogeneity
**Weight, kg**	7	-0.78	-1.69~0.13	0.06	82	<0.0001
**Study duration**						
< 12weeks	4	-0.96	-3.29～1.37	0.42	82	0.0008
≥12 weeks	3	-0.79	-1.70～0.11	0.09	77	0.01
**Study population**						
Asian	3	-0.31	-1.66～1.04	0.65	51	0.13
Western	4	-1.12	-2.51～0.11	0.07	87	0.0007
**Language**						
Without Chinese	6	-0.83	-1.75～0.09	0.08	86	<0.0001
**BMI, kg/m^2**	5	-0.81	-1.69~0.07	0.07	83	0.0001
**Study duration**						
< 12weeks	2					
≥12 weeks	3	-0.58	-2.55～1.38	0.56	90	<0.0001
**Study population**						
Asian	3	-0.59	-2.19～1.01	0.47	90	<0.0001
Western	2					
**Language**						
Without Chinese	3	-0.42	-2.06～1.21	0.61	89	<0.0001
**FPG, mg/dL**	6	2.33	0.63~4.03	0.007	10	0.35
**Study duration**						
< 12weeks	3	3.19	0.97~5.42	0.005	0	0.42

≥12 weeks	3	0.39	-5.39～6.17	0.89	47	0.15
**Study population**						
Asian	3	-2.54	-7.71～1.64	0.34	0	0.46
Western	3	2.92	1.12～4.72	0.001	0	0.91
**Language**						
Without Chinese	4	2.68	0.93～4.42	0.003	0	0.72
**FINS, mIU/ml**	7	-2.69	-3.81～-1.57	<0.0001	46	0.08
**Study duration**						
< 12weeks	3	-2.04	-4.27～0.20	0.07	30	0.13
≥12 weeks	4	-3.04	-4.56～-1.51	<0.0001	56	0.08
**Study population**						
Asian	4	-2.88	-5.02~-0.73	0.009	40	0.17
Western	3	-2.49	-4.73～-0.24	0.003	68	0.05
**Language**						
Without Chinese	6	-2.41	-3.60～-1.21	<0.0001	54	0.07
**HOMA-IR**	6	-0.41	-0.80～-0.02	0.04	94	<0.00001
**Study duration**						
< 12weeks	3	-0.83	-1.58～-0.09	0.03	47	0.15
≥12 weeks	3	-0.20	-0.65～0.25	0.38	97	<0.00001
**Study population**						
Asian	3	-0.53	-1.26~0.20	0.16	45	0.16
Western	3	-0.37	-1.10~0.35	0.31	80	0.007
**Language**						
Without Chinese	5	-0.34	-0.73～0.06	0.10	95	<0.00001
**TG, mg/dl**	6	-17.13	-35.37～1.11	0.07	88	<0.00001
**Study duration**						

< 12weeks	3	-31.38	-41.77～-21.0	<0.0001	0	0.64
≥12 weeks	3	-2.70	-14.45～9.05	0.65	44	0.17
**Study population**						
Asian	2					
Western	4	-24.76	-33.67～-15.85	<0.0001	56	0.08
**Language**						
Without Chinese	5	-15.46	-34.97～4.05	0.12	90	<0.00001
**TC, mg/dl**	6	-10.68	-24.57～3.21	0.13	94	<0.00001
**Study duration**						
< 12weeks	3	-18.80	-38.62~1.02	0.06	59	0.09
≥12 weeks	3	-1.38	-11.36～8.60	0.79	85	0.002
**Study population**						
Asian	2					
Western	4	-9.07	-35.31～17.17	0.50	96	<0.00001
**Language**						
Without Chinese	5	-8.02	-22.67～6.61	0.28	95	<0.00001
**HDL-C, mg/dl**	6	1.94	-1.82～5.69	0.31	95	<0.00001
**Study duration**						
< 12weeks	3	-2.05	-7.44～3.34	0.46	37	0.21
≥12 weeks	3	4.94	-0.64～10.51	0.08	91	<0.0001
**Study population**						
Asian						
Western	4	1.83	-5.98～9.64	0.65	95	<0.00001
**Language**						
Without Chinese	5	1.03	-2.76～4.81	0.59	95	<0.00001
**TT, nmol/L**	5	-0.22	-0.62～0.18	0.29	85	<0.0001

**Study duration**						
< 12weeks	3	-0.26	-0.72～0.19	0.25	80	0.006
≥12 weeks	2					
**Study population**						
Asian	3	-0.38	-1.06~0.30	0.28	79	0.07
Western	2					
**Language**						
Without Chinese	4	-0.24	-0.66～0.18	0.26	88	<0.0001
**FAI**	5	-0.05	-0.79～0.7	0.90	71	0.003
**Study duration**						
< 12weeks	2					
≥12 weeks	3	0.37	-0.84～1.57	0.55	29	0.24
**Study population**						
Asian	3	0.24	-0.36~0.84	0.43	75	0.02
Western	2					
**Language**						
Without Chinese	4	-0.51	-2.40～1.39	0.60	71	0.02
**SHBG, nmol/L**	4	6.20	-2.90～15.29	0.22	91	<0.00001
**Study duration**						
< 12weeks	2					
≥12 weeks	2					
**Study population**						
Asian	3	11.86	-5.52~29.25	0.18	94	<0.00001
Western	1					
**Language**						
Without Chinese	3	-0.90	-3.96～2.14	0.56	43	0.17

Without Chinese	3	-0.90	-3.96～2.14	0.56	43	0.17

BMI, body mass index; DHEAS, dehydroepiandrosterone sulfate; FAI, free androgen index; FINS, fasting insulin; FPG, fasting plasma glucose; HDL-c, high density lipoprotein cholesterol; HOMA-IR, homeostasis model assessment-insulin resistance; LDL-C, low density lipoprotein cholesterol; SHBG, sex hormone-binding globulin; TC, total cholesterol; TG, triglyceride; TT, total testosterone

Supplementary Table 4. Risk of bias assessment in each study

Author, year (reference)	Allocation sequence generation	Allocation concealment	Blinding participants and personnel	Blinding outcome assessment	Incomplete outcome reporting	Selective outcome reporting	Other sources of bias
Toscani *et al.*, 2011 [37]	Unclear	Low	Unclear	Low	Low	Unclear	Low
Stamets *et al.*, 2004 [38]	Low	Unclear	Unclear	Low	High	Low	Low
Moran *et al.*, 2003 [40]	Unclear	Low	High	Low	High	Unclear	High
Mehrabani *et al.*, 2012 [41]	Unclear	Low	Low	Low	High	Low	Low
Nadjarzadeh et al., 2021 [44] and Elham HM et al., 2020 [42]	Low	Low	Low	Low	Low	Unclear	Low
Kasim-Karakas *et al.*, 2009 [43]	Unclear	Low	Low	Low	High	Low	Low
Dou *et al.*, 2023 [45]	Low	Unclear	High	Low	High	Unclear	Low
Chen *et al.*, 2021 [46]	Low	Unclear	High	Low	Low	Unclear	Unclear

References

37. Toscani MK, Mario FM, Radavelli-Bagatini S, Wiltgen D, Matos MC, Spritzer PM. Effect of high-protein or normal-protein diet on weight loss, body composition, hormone, and metabolic profile in southern Brazilian women with polycystic ovary syndrome: a randomized study. Gynecol Endocrinol. 2011 Nov;27(11):925-30. doi: 10.3109/09513590.2011.564686
38. Stamets K, Taylor DS, Kunselman A, Demers LM, Pelkman CL, Legro RS. A randomized trial of the effects of two types of short-term hypocaloric diets on weight loss in women with polycystic ovary syndrome. Fertil Steril. 2004 Mar;81(3):630-7. doi: 10.1016/j.fertnstert.2003.08.023
39. Moran LJ, Noakes M, Clifton PM, Norman RJ. The effect of modifying dietary protein and carbohydrate in weight loss on arterial compliance and postprandial lipidemia in overweight women with polycystic ovary syndrome. Fertil Steril. 2010 Nov;94(6):2451-4. doi: 10.1016/j.fertnstert.2010.02.057.
40. Moran LJ, Noakes M, Clifton PM, Tomlinson L, Galletly C, Norman RJ. Dietary composition in restoring reproductive and metabolic physiology in overweight women with polycystic ovary syndrome. J Clin Endocrinol Metab. 2003 Feb;88(2):812-9. doi: 10.1210/jc.2002-020815.
41. Mehrabani HH, Salehpour S, Amiri Z, Farahani SJ, Meyer BJ, Tahbaz F. Beneficial effects of a high-protein, low-glycemic-load hypocaloric diet in overweight and obese women with polycystic ovary syndrome: a randomized controlled intervention study. J Am Coll Nutr. 2012 Apr;31(2):117-25. doi: 10.1080/07315724.2012.10720017.
42. Elham HM, Akram G-A, Nahid R-J, Mohammad M, Nooshin A, Seyedeh MN, et al. Effect of fennel supplementation along with high-protein, low-carbohydrate weight-loss diet on insulin resistance and percentage of fat and muscle mass in overweight/obese women with polycystic ovary syndrome. Journal of Functional Foods. 2020.103848. DOI:10.1016/j.jff.2020.103848.
43. Kasim-Karakas SE, Almario RU, Cunningham W. Effects of protein versus simple sugar intake on weight loss in polycystic ovary syndrome (according to the National Institutes of Health criteria). Fertil Steril. 2009 Jul;92(1):262-70. doi: 10.1016/j.fertnstert.2008.05.065.
44. Nadjarzadeh A, Ghadiri-Anari A, Ramezani-Jolfaie N, Mohammadi M, Salehi-Abargouei A, Namayande SM, Mozaffari-Khosravi H, Hosseini-Marnani E. Effect of hypocaloric high-protein, low-carbohydrate diet supplemented with fennel on androgenic and anthropometric indices in overweight and obese women with polycystic ovary syndrome: A randomized placebo-controlled trial. Complement Ther Med. 2021 Jan;56:102633. doi: 10.1016/j.ctim.2020.102633
45. Dou P, Zhang TT, Xu Y, Xue Q, Shang J, Yang XL. [Effects of three medical nutrition therapies for weight loss on metabolic parameters and androgen level in overweight/obese patients with polycystic ovary syndrome. Zhonghua Yi Xue Za Zhi. 2023 Apr 11;103(14):1035-1041. Chinese. doi: 10.3760/cma.j.cn112137-20220930-02066.

46. Chen DM, Yang L, Jiang JC. The effects of high protein diet on glucolipid metabolism and BMI level in obese patients with polycystic ovary syndrome. Jiceng Yi Xue Lun Tan. 2021, 25(14):3.

a. the NIH 1990 criteria: Zawadski JK, Dunaif A. Diagnostic criteria for polycystic ovary syndrome: Towards a rational approach. In: Dunaif A, Givens JR, Haseltine F, editors. Polycystic Ovary Syndrome. Boston, MA: Black-well Scientific Publications; 1992. p. 377–84.

b. Rotterdam 2003 criteria: The Rotterdam ESHRE/ASRM-sponsored PCOS Consensus Workshop Group. Revised 2003 consensus on diagnostic criteria and long-term health risks related to polycystic ovary syndrome (PCOS). Hum Reprod. 2004;19(1):41-7.

c. AEPCOS 2006 criteria: Azziz R, Carmina E, Dewailly D, Diamanti-Kandarakis E, Escobar-Morreale HF, Futterweit W, Janssen OE, Legro RS, Norman RJ, Taylor AE, et al. The Androgen Excess and PCOS Society criteria for the polycystic ovary syndrome: The complete task force report. Fertil Steril. 2009;91(2):456
